# Forecasting Dengue: Evaluating the Role of Hydroclimate Information in Subseasonal to Seasonal Prediction

**DOI:** 10.1029/2024GH001325

**Published:** 2025-09-01

**Authors:** Maxwell R. W. Beal, Jorge Osorio, Karl Ciuoderis, Juan Pablo Hernandez‐Ortiz, Paul Block

**Affiliations:** ^1^ Department of Civil and Environmental Engineering University of Wisconsin‐Madison Madison WI USA; ^2^ Now at Ocean Ecology Laboratory NASA Goddard Space Flight Center/Science Systems and Applications, Inc Greenbelt MD USA; ^3^ Department of Pathobiological Sciences Global Health Institute School of Veterinary Medicine University of Wisconsin‐Madison Madison WI USA; ^4^ GHI One Health Colombia and One Health Genomic Laboratory Medellín Colombia; ^5^ Facultad of Life Sciences Universidad Nacional de Colombia, Sede Medellín Medellín Colombia

**Keywords:** dengue, forecast, hydroclimate, seasonal, vector‐borne disease, global health

## Abstract

Dengue fever is a mosquito‐borne viral disease rapidly creating a significant global public health burden, particularly in urban areas of tropical and sub‐tropical countries. Hydroclimatic variables, particularly local temperature, precipitation, relative humidity, and large‐scale climate teleconnections, can influence the prevalence of dengue by impacting vector population development, viral replication, and human‐mosquito interactions. Leveraging predictions of these variables at lead times of weeks to months can facilitate early warning system preparatory actions such as allocating funding, acquisition and preparation of medical supplies, or implementation of vector control strategies. We develop hydroclimate‐based statistical forecast models for dengue virus (DENV) at 1‐, 3‐, and 6‐ month lead times for four cities across Colombia (Cali, Cúcuta, Medellín, and Leticia) and compare with standard autoregressive models conditioned on dengue case counts. Our results indicate that (a) hydroclimate‐based models are particularly skillful at 3‐ and 6‐ month lead times when autoregressive models often fail, (b) sea surface temperatures are the most skillful predictor at 3‐ and 6‐ month leads and (c) application of hydroclimate models are most beneficial when average DENV incidence is low, autoregressive relationships are weak, but outbreaks may still occur.

## Plain Language Summary

Dengue fever is a mosquito‐borne viral disease rapidly creating a significant global public health burden, particularly in urban areas of tropical and sub‐tropical countries. Temperature, precipitation, and other climate variables have established relationships with mosquito populations and replication of dengue virus (DENV). Using climate variables to predict dengue cases at longer (weeks to months) lead times may help improve preparation for dengue outbreaks, such as allocating funding, acquisition and preparation of medical supplies, or implementation of vector control strategies. We develop forecasts for DENV at 1‐, 3‐, and 6‐ month lead times for four cities across Colombia (Cali, Cúcuta, Medellín, and Leticia). Our results show that climate information is most useful for dengue forecasts at 3‐ and 6‐ month lead times. We also find that climate‐based predictions are beneficial in scenarios where dengue incidence is typically low, but outbreaks may still occur.

## Key Points

- We develop hydroclimate‐based dengue forecasts at 1‐, 3‐, and 6‐ month leads
- Hydroclimate‐based models are particularly skillful at 3‐ and 6‐ month lead times
- Models are most beneficial when mean incidence is low but outbreaks may still occur

## 1 Introduction

Vector‐borne diseases are a significant contributor to morbidity and mortality, globally (Hunter, 2003). Climate and hydrology play a critical role in the emergence of vectors and pathogens. In many places, environmental constraints on vector and pathogen development result in notable inter‐ and intra‐ annual variability in vector‐borne disease burden (Yuan et al., 2020). Climate and hydrology have complex and often contrasting effects on vector and pathogen biology that have been shown to influence the spread of disease. Pathogen and vector development have been shown to increase with temperature, but responses are often species and location‐specific (Whiting et al., 2001). Precipitation may increase habitat availability through ponding but may eliminate vectors in extreme precipitation events (Whiting et al., 2001). The complexity of hydroclimate impacts on vectors and pathogens means establishing robust relationships between hydroclimate variables and infectious disease case counts remains challenging. Identifying and associating the nuances of these and other environmental drivers of vector and pathogen development can facilitate the identification of conditions associated with high disease burden, ideally leading to an increased understanding and development of early warning systems. Skillful prediction of vector‐borne disease incidence and severity may promote public health preparedness, particularly at longer timescales. Recently, increasing focus has been placed on predicting hydroclimate conditions at timescales between short‐range weather predictions and long‐range seasonal outlooks. This subseasonal‐to‐seasonal (S2S) scale has been identified as a key timeframe for decision‐making in many sectors (White et al., 2017). Public health may benefit from S2S forecasts by informing decisions that require long lead times, such as allocating funding, acquisition and preparation of medical supplies, or implementation of vector control strategies (Brunet et al., 2010), ideally leading to reduced community morbidity and mortality and costs for relief organizations.

Among vector‐borne diseases, dengue stands out as a global public health threat (Guzman et al., 2010). Recent years have marked record dengue outbreaks. Over 10 million cases were reported in 2024, concentrated in the Americas (Lancet, 2024). Dengue has spread significantly since its re‐emergence in Latin America, with cases rising rapidly since the 1980's (Lenharo, 2023). On 18 June 2024, the Pan American Health Organization released a document detailing a record number of dengue cases during the first half of the year, exceeding the maximum number of cases historically reported in any year (Pan American Health Organization, 2024). Dengue virus (DENV) is spread to humans through the bite of infected *Aedes* mosquitoes and is considered a largely urban disease. Epidemics may become more frequent as the population of Latin America increases, and urbanization enhances opportunities for transmission (Gubler, 2011). Like other vector‐borne diseases, the incidence of DENV has been linked to climate, including local‐scale metrological variables that can modulate vector habitat, survival, and pathogen replication (Duarte et al., 2019; Me et al., 2018; Villegas et al., 2020).

Among Latin American counties, Colombia is a strong candidate for developing targeted S2S forecasts of DENV incidence. Colombia is experiencing a resurgence of vector‐borne diseases and has been identified as an emerging disease hotspot (Jones et al., 2008), despite the presence of a national integrated vector control strategy. All four DENV serotypes are actively circulating in many parts of the country, and there has been a significant increase in the number of severe DENV cases since re‐emergence (Gutierrez‐Barbosa et al., 2020; Ocampo et al., 2014). Severe outbreaks in 2013 and 2019 prompted requests from The Red Cross Society of Colombia to the Red Cross Disaster Relief Emergency Fund (DREF) for funds to support dengue interventions (“2013 DREF Final Report Colombia: Dengue Outbreak,” 2013; “2019 DREF Final Report Colombia: Dengue Outbreak,” 2019). Both scenarios involved widespread dengue outbreaks at hyper‐endemic levels, with high caseloads persisting for weeks to months. These funds often support interventions after the outbreak has occurred, rather than prior to help support preparedness. Ideally, information on expected dengue incidence at longer lead times can support public health planning and staging of resources, reducing costs and morbidity/mortality. Click or tap here to enter text. The Colombian government is currently developing a Climate and Health early action protocol. Early actions will be based on 1‐month lead forecasts from Colombia's national health agency (INS) and the national meteorological agency (IDEAM), however, longer lead times (>1 month) are currently not included in the protocol due to higher uncertainties in traditional dengue models at these lead times. Evaluating the ability of climate information to extend dengue forecast lead times will complement current efforts.

While the influence of local hydrology and climate on vector‐borne disease is well documented in tropical areas, including Colombia (Cano‐Pérez et al., 2022; E. Muñoz et al., 2021; Ordonez‐Sierra et al., 2021) these variables can have interacting and contrasting effects on DENV transmission that are difficult to capture, particularly on S2S timescales. Colombia frequently experiences hydrologic extremes, often driven by large climate cycles, like the El Nino Southern Oscillation (ENSO) (Poveda et al., 2011; Waylen & Poveda, 2002). Significant portions of the country are favorable to transmission of vector‐borne disease (Cabrera & Selvaraj, 2020), making the region a suitable study site for the investigation of hydroclimate‐vector relationships. Additionally, spatial variability in hydroclimate variables across Colombia is significant. Differences in climate and land cover across Colombia may have impacts on hydroclimate‐disease relationships that might be reflected in model development. Finally, Colombia's National Public Health Surveillance System (SIVIGILA) has a sufficiently long time series for the development of data‐driven forecasts and modeling (Eastin et al., 2014; Zhao et al., 2020).

This paper focuses on developing tailored statistical forecast models for DENV at 1‐, 3‐, and 6‐ month lead times for four diverse cities across Colombia (Cali, Cúcuta, Medellín, and Leticia). While several climate‐based S2S warning systems have been developed for facets of DENV transmission (Á. G. Muñoz et al., 2020; Tompkins et al., 2019), relatively little work has been done in Colombia. Using DENV case data from SIVIGILA, S2S forecast models are developed and evaluated using local scale and global scale hydroclimate variables. For each lead time, statistical model predictions are compared with an autoregressive model, the traditional approach for dengue prediction.

Specifically, we investigate the following questions:

1. How well do local and global hydroclimate variables correlate with DENV case load at 0‐, 1‐, 3‐, and 6‐ month lead times?
2. Can hydroclimate variables improve forecast performance over climatological and autocorrelation models and how does this vary with lead time?
3. Under what DENV conditions (high, low case load) are hydroclimate variables most useful for prediction?

This modeling approach aims to better understand the dominant hydroclimatic drivers of DENV in Colombia at concurrent to seasonal lead times and leverage those relationships to develop forecast systems to inform public health decision making. Forecast development provides an opportunity to investigate the role of climate information in DENV early warning systems broadly at different S2S scales and may provide insight into the conditions under which climate‐based early warning systems are valuable for DENV preparedness.

## 2 Methods

### 2.1 Study Site

Colombia has identified DENV as a significant public health threat since the 1950's. The suspension of vector control campaigns targeting *Aedes* mosquitos in 1970 led to a resurgence of DENV infections that persists today (Gutierrez‐Barbosa et al., 2020). Most cases occur in urban areas of Colombia, driven in part by high population density and water infrastructure that may act as breeding sites for *Aedes aegypti* (Villar et al., 2015). DENV is considered hyperendemic in Colombia, with co‐circulation of all four DENV serotypes. Several prevention measures are available to address and prevent dengue outbreaks. A dengue vaccine, Dengvaxia, is currently available but not yet implemented in Colombia. The vaccine is recommended only for people with confirmed previous dengue infection (WHO, 2019). Other effective prevention measures include the use of larvicide, insecticide (e.g., spraying, treated nets), and personal preventative measures to avoid mosquito bites (Ocampo et al., 2014; Sepulveda & Vasilieva, 2016). Despite the availability of Dengvaxia, recent work suggests that traditional vector prevention strategies in Colombia remain a viable and cost‐effective option (Claypool et al., 2021). Therefore, developing tools to inform the early warning and activation of common vector control strategies in Colombia may be advantageous for public health managers.

**Figure 1:**
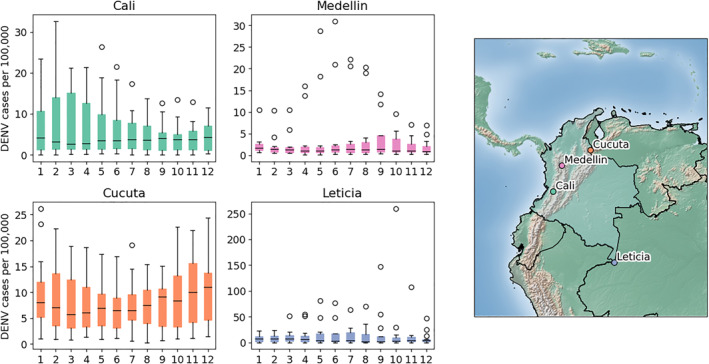
Boxplots of average monthly dengue virus incidence per 100,000 population in each study site location (left) and a map of study site locations in Colombia (right).

DENV case data is accessed from SIVIGILA run by the National Institutes of Health of Colombia (INS), for four cities including Cali, Cúcuta, Medellín, and Leticia. Case counts are reported by clinics and hospitals to insurance agencies and regional health authorities, which are then sent to INS for consolidation. Dengue cases are defined by SIVIGILA as all people with acute febrile illness (<7 days) with two or more of the following manifestations: headache, retro‐orbital pain, myalgia, arthralgia, or rash (Rico‐Mendoza et al., 2019). Case count data are available through 2021 in all cities. Data begin in 2006 in Cali and Cúcuta, 2008 in Leticia, and 2009 in Medellín. Population data is available for each city through 2020, allowing for an annual estimate of DENV incidence (Figure 1).

Each city expresses some interannual variability in case load, however, DENV case seasonality is notably different among cities. Further, while each city exhibits seasons in which the potential for increased DENV transmission appears higher, some years have relatively low DENV transmission across all months. Given the lack of a defined transmission season across cities, models are developed separately for each city and month (48 models in total), following a defined modeling structure.

### 2.2 Predictor Selection

Predictors (i.e., hydroclimate observations occurring before the month of interest) are selected based on a literature review and evaluated through a correlation analysis at 0‐, 1‐, 3‐, and 6‐ month lead times. Hydroclimate variables including temperature, precipitation, humidity, and large‐scale climate phenomena are potentially useful sources of predictive information given the wide availability of data products, temporal persistence of hydrology and climate variables (Barnston, 1994; Markowski & North, 2003), and the maturity of forecasting products for hydroclimate variables (Becker et al., 2014; Pegion et al., 2019). Correlations are calculated separately for each city and each month of the year (e.g., Cali February DENV incidence is correlated with Cali January temperature for the 1‐month lead time). Eight hydroclimate variables, outlined below, are evaluated based on their physical relationship with DENV incidence.

**Figure 2:**
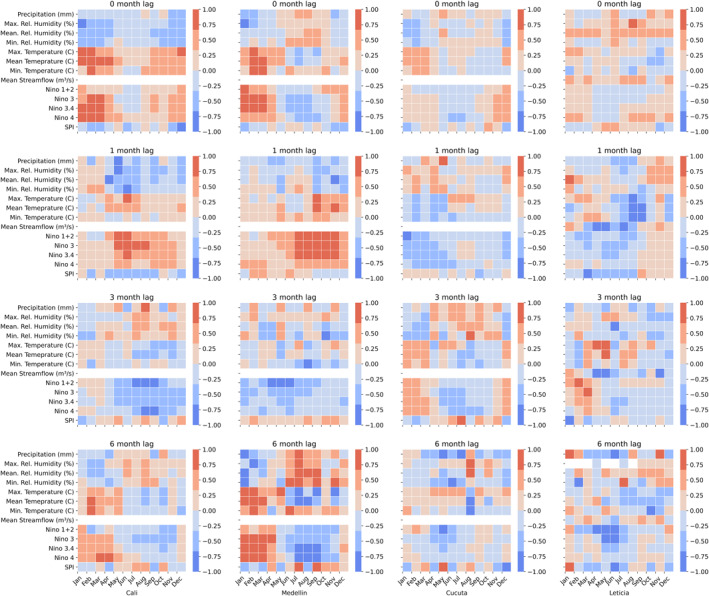
Results of a correlation analysis between dengue virus incidence and one‐dimensional candidate predictor variables for each study location (columns) and 0‐, 1‐, 3‐, and 6‐month lead times (rows).

As discussed above, temperature and precipitation have been widely shown to influence pathogen and vector development and dengue transmission at lead times from weeks to months (Johansson et al., 2009). Phenomena such as ENSO can influence vector‐borne disease by modulating local climate conditions (e.g., temperature, humidity, and precipitation) through atmospheric teleconnections (Barnston, 1994; Giannini et al., 2000; Markowski & North, 2003). The effects of ENSO on the climate and hydrology of Colombia are well‐established (Poveda et al., 2011) and have been previously linked directly to the incidence of vector‐borne disease in Colombia (E. Muñoz et al., 2021; Poveda et al., 2000). Given this established connection, indices for ENSO regions 1 + 2, 3, 3.4, and 4 are evaluated as candidate predictors of DENV incidence. Leticia is located on the banks of the Amazon River, thus streamflow is also considered as a candidate predictor, given the potential for the river to create vector habitat under certain flow conditions. These one‐dimensional (i.e., gauged data and climate indices) predictors are evaluated in a simple correlation analysis with DENV incidence at each of the relevant lead times (Figure 2). Predictors correlating with DENV incidence at the 95% confidence level are then retained as predictors for model development.

Colombia's climate and hydrology are also influenced by a variety of low‐level jets with origins in the Caribbean and coastal tropical Pacific (Salas et al., 2020). To capture the variable sources of large‐scale hydroclimate influences, regions of global sea surface temperature (SST) data are evaluated as potential predictors. While ENSO is known to influence climate and hydrology in Colombia, other climate phenomena may also have impacts. Global analysis of SSTs captures these regions of influence that are otherwise missed by using ENSO indices alone. Similarly, regions of geopotential height are known to play a significant role in atmospheric circulation patterns and may influence the distribution of weather across South America. One such feature is the Bolivian high; an upper‐level anticyclone that manifests as a high pressure region. The Bolivian high is thought to have significant impacts on temperature and precipitation across South America (Lenters & Cook, 1997). Therefore, regions of 200 mb geopotential height are used as candidate predictors.

A Monte Carlo analysis is used to identify SST and pressure regions that may serve as candidate predictors. Correlations between DENV incidence and each variable are calculated globally. The number of significant correlations is then compared to 1,000 trials in which the target variable is randomly shuffled (following Zimmerman et al., 2016). This process helps to remove spurious correlations, ultimately providing a more robust selection of global climate predictors. Ideally, this analysis captures regions of influence beyond traditional climate indices (Nino 3.4, Multivariate ENSO Index, etc.). This process is performed for each city in each month. For selected regions, a principal component analysis (PCA) is performed to extract the dominant signals. Principal components that explain more than 10% of the variability are retained as candidate predictors.

Autoregressive predictors (DENV incidence at 1‐, 3‐, and 6‐ month lags) are also evaluated as predictors for each lead time and month. Therefore, a total of nine predictors (eight hydroclimate and one autoregressive) are evaluated for each city, month, and lead time (Table 1).

**Table 1:** Variables Used in Correlation Analysis With Monthly Dengue Virus (DENV) Incidence at 0‐, 1‐, 3‐, and 6‐ Month Lead Times

Predictors (monthly)	Source	Resolution
Lagged DENV Incidence (cases/population/month)	SIVIGILA	City
Total Precipitation (mm)	IDEAM	Gauged
Standardized Precipitation Index (–)	IDEAM	Gauged
Relative Humidity (mean, max, min) (%)	IDEAM	Gauged
Temperature (mean, max, min) (°C)	IDEAM	Gauged
Streamflow (Leticia only) (m^3^/s)	DHIME‐IDEAM	Gauged
ENSO Regions (1 + 2, 3, 3.4, 4) (°C)	NOAA ERSST v5	2°
Global Sea Surface Temperature (°C)	NOAA ERSST v5	2°
Global Geopotential Height (200 mb) (gpm)	NCEP‐NCAR	2°

*Note.* Instituto de Hidrología, Meteorología y Estudios Ambientales (IDEAM), Datos de Hidrología y Meteorología (DHIME), National Oceanic and Atmospheric Association Extended Reconstructed Sea Surface Temperature Data set Version 5 (NOAA ERSST v5), National Centers for Environmental Prediction/National Center for Atmospheric Research (NCEP‐NCAR).

Evaluation at concurrent (0‐month lead) timescales are also included to assess whether *predictions* of hydroclimate variables from the North American Multi‐Model Ensemble (NMME) set of dynamical models may be skillful in predictions of DENV incidence. The NMME platform is a set of models that generates global forecasts with lead times up to 1 year (Kirtman et al., 2014). Here, only one model from the NMME system is presented, however, model codes are built to incorporate several NMME model systems. Adoption of multi‐model ensembles (i.e., running a set of numerical models with random perturbations in their initial conditions to simulate weather conditions for an upcoming season) generally improves collective accuracy compared with any single model. In this work, forecast outputs for temperature, precipitation, and sea surface temperatures are taken from the NOAA Geophysical Fluid Dynamics Laboratory's Seamless System for Prediction and Earth System Research (GFDL‐SPEAR).

### 2.3 Modeling Approach

Model hindcasts for DENV are developed for 1‐, 3‐, and 6‐ month lead times in the four cities. Given the variability in seasonality of DENV incidence across cities, unique models are developed for each month. For example, a model is developed to predict January DENV incidence in Cali, leveraging predictors from 1‐, 3‐, and 6‐ months prior (i.e., forecasts issued 31 December, 31 October, and 31 July, respectively). This approach allows for evaluation of model skill and climate information importance in each month.

DENV incidence is predicted with a random forest regression model, implemented using the scikit‐learn package in Python (Pedregosa et al., 2011). Random forests are a non‐parametric ensemble learning method based on constructing many decision trees, each collectively voting on an outcome (Breiman, 2001). Random forests are well‐suited to modeling DENV incidence given the non‐normal distribution nature of the data. Additionally, random forests are more robust to overfitting compared to alternative deep learning methods. This is a particularly important feature given the limited size of data sets. Random forests also benefit from computing variable importance (marginal decrease in model performance when each variable is excluded) during the modeling process. This allows for directly assessing climate variable importance among cities, lead times, and months. A leave one out cross validation (LOOCV) approach is taken to evaluate model performance. For each city and month specific model, 1 year of the timeseries is dropped, the model is constructed, and the missing value is predicted. This approach is conducted iteratively until all years have been predicted, allowing for an estimation of model skill. LOOCV is useful for evaluating model performance in a data limited environment. Uncertainty in model outputs is estimated using the forestci package, which implements a Monte‐Carlo based estimation of variance for random forest regression (Wager et al., 2014). In addition to the random forest model, PCA and negative binomial regression models are evaluated. Negative binomial models are commonly applied to over dispersed count data and are often used for modeling of incidence data (Abiodun et al., 2019; Evans & Adenomon, 2014; Hilbe, 2011). A PCA is performed on each predictor set before constructing the negative binomial model. PCA removes the effects of multi‐collinearity, reducing artificial inflation of predictive skill. Principal components are retained for the model if they explain more than 10% of the variability in the predictor set. Negative binomial models are constructed using R version 4.2.1.

An autoregressive reference model, commonly used for disease transmission forecasting (Baharom et al., 2022), is constructed for 1‐, 3‐, and 6‐ month lead times for each month in each city using linear regression to compare against each random forest model.

### 2.4 Model Evaluation

Models are evaluated with deterministic, probabilistic, and categorical metrics. The coefficient of determination (*R* ^2^) and root mean squared error (RMSE) are used to evaluate deterministic model performance. Accuracy, sensitivity, and specificity are evaluated to determine categorical performance. The continuous ranked probability score (CRPS) is also evaluated for an ensemble (probabilistic) version of the forecast, using the *properscoring* package (version 0.1, available at https://pypi.org/project/properscoring/) in python. The CRPS generalizes mean absolute error to a distributional prediction, comparing an ensemble forecast to the observed value (Epstein, 1969; Hersbach, 2000) (Equation 1).

(1) $$\text{CRPS}\left(F,y_{\text{obs}}\right)={\int_{-\infty }^{\infty }\left[F\left(y\right)-H\left(y-y_{\text{obs}}\right)\right]}^{2}dy$$

Where F(y) is the cumulative density function of the forecast, $y_{\text{obs}}$ is the observed value, and $H\left(y-y_{\text{obs}}\right)$ is the Heaviside step function (Equation 2).

(2) $$H\left(y\right)=\left\{\begin{array}{c} 0\;\text{for}\;y<0\\ 1\;\text{for}\;y\geq 0 \end{array}\right.$$

The CRPS can be normalized to a reference forecast to obtain the continuous ranked probability skill score (CRPSS). This score ranges from −∞ to 1, where 0 represents no skill, 1 represents a perfect forecast, and negative values indicate a forecast inferior to the reference. Here, the reference forecast is defined as the observed distribution dengue incidence across all years, specific to each city and month.

(3) $$\text{CRPSS}=1-\frac{{\text{CRPS}}_{\text{forecast}}}{{\text{CRPS}}_{\text{reference}}}$$

Ensemble predictions for each year in the hindcast are based on errors, defined as the difference between predicted and observed dengue incidence in the leave‐one‐out cross‐validation approach. Errors are fit to a normal distribution, with mean zero, using a maximum likelihood estimation. For each hindcast year, 100 random draws from the distribution are added to the deterministic forecast to form the ensemble (Helsel & Hirsch, 1992).

Dengue incidence categories are defined using the endemic channel, a tool to estimate the central tendency and upper and lower limits of epidemiological data (Bortman, 1999). The endemic channel calculation used here is adapted from the R package epiCo, a software package developed specifically for the evaluation of vector‐borne diseases in Colombia (Umaña et al., 2024). An endemic channel is calculated for each city at a monthly interval. The central tendency is calculated for each month by taking the geometric mean of historical case data for each month. The lower and upper limits are then calculated using the geometric standard deviation, representing a 95% confidence interval. Epidemic years are commonly removed from the calculation of the endemic channel to provide a better estimate of “normal” disease conditions. Here, monthly case counts are omitted from the calculation if they exceed the 90th percentile of observed cases in each city. The deterministic model results are binned into the four categories represented by the endemic channel: *Below Safety*, *Above Safety*, *Warning*, and *Epidemic.* These categories are then used to evaluate the accuracy, sensitivity, and specificity of each model at 1‐, 3‐, and 6‐ month lead times.

Predictor variable importance is also assessed for each model. Variable importance is calculated as the mean decrease impurity, calculated for each feature as the total decrease in node impurity weighted by the probability of reaching that node, averaged over all trees. Variable importance is assessed for each model. For comparison, variable importance is then grouped into categories based on variable type and source to better understand the influence of different processes (Table 2).

**Table 2:** Predictor Categories for Variable Importance Assessment

Source category	Category type	Predictor
Autocorrelation	Autocorrelation	DENV Incidence
Lagged	Hydrology	Total Precipitation (mm)
Lagged	Hydrology	Standardized Precipitation Index (–)
Lagged	Hydrology	Relative Humidity (mean, max, min) (%)
Lagged	Hydrology	Streamflow (Leticia only) (m^3^/s)
Lagged	Temperature	Temperature (mean, max, min) (°C)
Lagged	Global Climate	ENSO Regions (1 + 2, 3, 3.4, 4) (°C)
Lagged	Global Climate	Global Sea Surface Temperature (°C)
Lagged	Global Climate	Global Geopotential Height (200 mb) (gpm)
NMME	Temperature	GFDL SPEAR Total Precipitation (mm)
NMME	Temperature	GFDL SPEAR Temperature (max, min) (mm)
NMME	Global Climate	GFDL SPEAR ENSO Regions (1 + 2, 3, 3.4, 4) (°C)

The LOOCV process creates a model structure corresponding to each month of DENV incidence data. Variable importance is calculated for each model realization in the LOOCV process. Variable importance can then be compared among lead times, cities, months, and endemic channel categories to answer questions regarding the role of climate information in predicting DENV incidence under a wide variety of conditions.

## 3 Results

### 3.1 Leading Predictors

Predictor variable relevance is evaluated two ways: the number of times a predictor is retained for a model (expressed as a percent), and the mean decrease in impurity (variable importance) calculated for a predictor when it is included in the random forest model structure.

The three most retained predictors across all cities, months, and lead times, with an importance score greater than zero are autocorrelation (lagged number of cases), the first principal component of lagged sea surface temperatures, and lagged Nino 3 and 3.4 regions (Table 3). This similarity in the most common predictors indicates the outsized importance of both autocorrelation and large‐scale climate features in prediction of DENV incidence. Notably, the percent inclusion and average importance vary for these predictors among lead times. At lead times of 1‐month, autocorrelation dominates. One month lagged DENV incidence is included as a predictor in 100% of models and on average accounts for 56% of the mean decrease in impurity (variable importance). While SST PC1 and Nino 3 SSTs are the next most important variables, they appear in only 35% and 23% of models, respectively, and have an average importance of less than 10%. As expected, the influence of autocorrelation declines for the 3‐ and 6‐ month lead times, and is included in only 69% and 27% of models, respectively. While the percent inclusion of SST and Nino predictors remains relatively similar at 3‐ and 6‐ month leads, average variable importance increases notably for both, as importance of autocorrelation declines. This generally indicates a shift in predictive power from autoregressive variables to large scale climate features as lead time increases.

**Table 3:** Three Most Retained Predictors for Each Lead Time, Across all Cities and Months With Importance Scores Greater Than Zero

Lead time	Predictor	Inclusion (*N* = 48) (%)	Average importance (%)
1‐month	Autocorrelation	100	56
	Sea Surface Temperature PC1	39.6	9
	Nino 3, 3.4 (1‐mo lag)	22.9	1
3‐month	Autocorrelation	75	45
	Sea Surface Temperature PC1	47.9	19
	Nino 3, 3.4 (3‐mo lag)	29.2	2
6‐month	Sea Surface Temperature PC1	45.9	25
	Autocorrelation	33.3	13
	Nino 3, 3.4 (6‐mo lag)	33.3	7.8

*Note.* Nino 3 and 3.4 are tied for inclusion at each lead time.

The most prevalent predictor variable types (Table 2) reflect the patterns indicated by the top individual predictors. At a 1‐month lead time, 100% of models include autoregressive predictors and 70.8% include global climate, followed by temperature predictors and hydrology predictors. At the 3‐month lead, autoregressive and global climate predictors are included in the same percentage of models. At the 6‐month lead time, the inclusion of global climate predictors surpasses autoregressive predictors 67%–27%. Temperature and hydrology predictors are ranked third and fourth, respectively, at all lead times.

**Figure 3:**
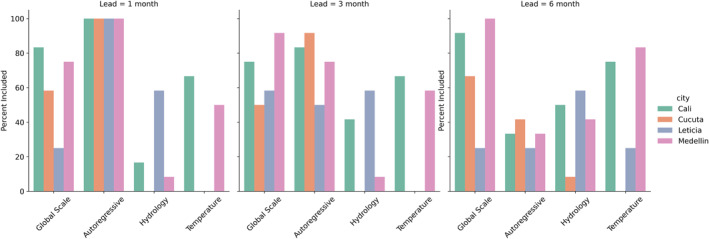
Percent of models for which each predictor type is included, by city and lead time.

The patterns for predictors included by city are generally similar to overall results, with some notable exceptions. In all cities, autoregressive predictors decline in percent inclusion and global climate features tend to increase with lead times (Figure 3). Local temperature and hydrologic predictors are generally included in fewer models. Models in Leticia use hydrologic predictors more frequently than in other cities. This is largely driven by max and mean relative humidity in Leticia, which is retained for several months of the year at all lead times. The use of hydrologic predictors in Cali and Medellín increases at the 6‐month lead time. Six‐month lagged mean and max relative humidity appear to be the most commonly used predictors for these cities, but lagged precipitation and the standardized precipitation index are included in some models as well.

Temperature predictors are included more often in Cali and Medellín models compared to Leticia and Cúcuta. These differences persist across lead times. Lagged temperature and NMME forecasts of maximum and minimum temperature are used frequently in Cali and Medellín models. Temperature is used infrequently in Cúcuta and Leticia, possibly due to higher average temperatures throughout the year. Average temperatures in Cúcuta and Leticia are roughly 28 and 26°C, respectively (Cali 25°C, Medellín 22°C). Therefore, temperature may not be a limiting factor for vector development in Cúcuta and Leticia. Further, this pattern is also likely related to better performance of global scale predictors in Cali and Medellín, as temperature predictors generally correlate highly with ENSO indices in these locations.

### 3.2 Model Performance

At the 1‐month lead time, all 48 city and month ‐specific models have at least one significantly correlating predictor with which to construct a random forest model. At 3‐month and 6‐month leads, 47 and 42 models are constructed. Months without a significantly correlating predictor default to a naïve forecast using the long‐term average of DENV incidence. Model skill is evaluated in a point‐by‐point comparison (deterministic), an ensemble comparison (probabilistic), and a categorical comparison (categorically).

*R* ^2^ and RMSE values are calculated for each city and month‐specific model and are compared to an autoregressive model. In some cases, autoregressive models are particularly poor resulting in negative *R* ^2^ values. In these cases, comparing climate‐based model performance against autoregressive models can inflate the gain in skill. Therefore, to be conservative, the *R* ^2^ values for the null models are restricted from zero to one. This autoregressive model with a minimum *R* ^2^ of 0 is referred to as the reference model below.

**Figure 4:**
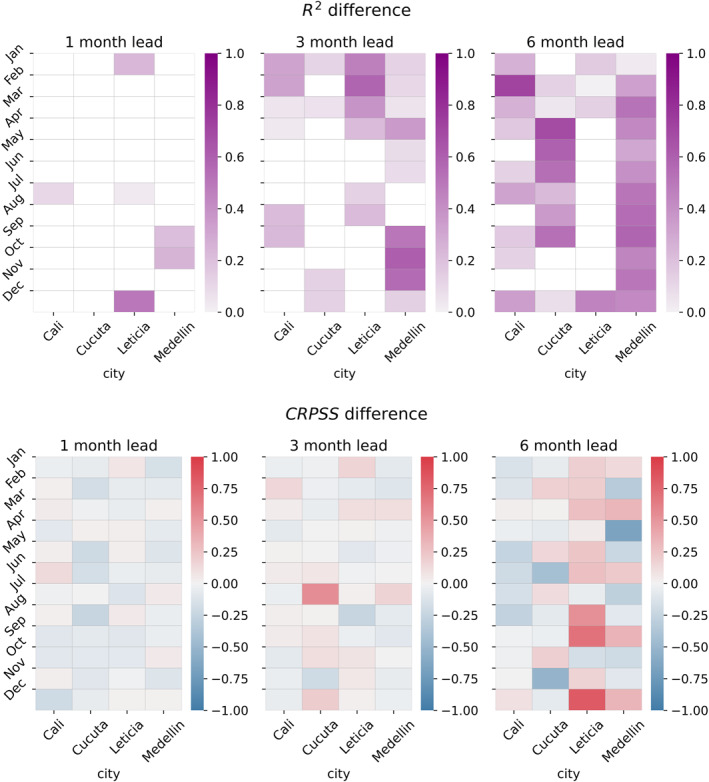
Improvements in random forest *R* ^2^ (coefficient of determination) and root mean squared error over reference models.

At the 1‐month lead only six (12.5%) models show improvement over reference model *R* ^2^ values (Figure 4); 25 (54%) models improve over reference models at the 3‐month lead time and 33 (70%) models improve over reference models at the 6‐month lead. For random forest models that showed improvement over reference models, the average increase (decrease) in *R* ^2^ (RMSE) of random forest models over reference models is 0.22 (−1.26) at the 1‐month lead, 0.24 (−1.87) at the 3‐month lead, and 0.34 (−1.94) at the 6‐month lead.

On average, improvements in forecast skill are greatest at the 6‐month lead and the largest proportion of climate‐based models show improvement over reference models. While gains at the 3‐month lead are more modest, performance over the reference models can be seen, particularly at the beginning and end of the year. Consistent improvements are seen across the year at the 6‐month lead time with the exception of Leticia. This is likely because global scale climate predictors dominate at the 6‐month lead in other cities but show little skill in Leticia.

Negative binomial models improve over reference models in fewer cases than random forest models do (8.3%, 12.5%, and 31.3% at the 1‐ 3‐, and 6‐ month lead times), however, they tend to have higher improvements in *R* ^2^ scores on average (0.11, 0.27, and 0.45 at the 1‐, 3‐, and 6‐ month leads). Nonetheless, the lack of consistent improvement across months is a notable weakness in the negative binomial models.

**Figure 5:**
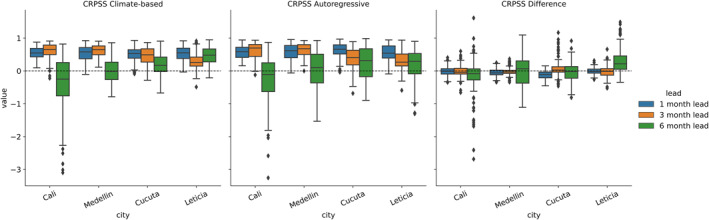
Continuous ranked probability skill score (CRPSS) by city and lead time for the climate‐based forecast, the autoregressive model, and the difference (CRPSS Climate‐based—CRPSS Autoregressive). CRPSS is calculated for each timestep (monthly).

The CRPSS provides a measure of ensemble forecast performance and is evaluated for the climate‐based forecast and the autoregressive model. Forecast performance for both the climate‐based forecast and the autoregressive model tend to decline from 1‐ to 6‐month lead times (Figure 5). Climate‐based models achieved median CRPSS values of 0.56, 0.53, and 0.18 at the 1‐, 3‐, and 6‐ month leads. CRPSS shows climate‐based models underperforming autoregressive models at the 1‐ and 3‐ month lead times, and outperforming at the 6‐month lead.

Forecast accuracy, sensitivity, and specificity are also calculated for each city, lead time, and category derived from the endemic channel. Accuracy represents the proportion of predicted categories that match observed categories. Sensitivity (true positive rate) measures how often a category is correctly predicted, conditioned on the total number of times that category was observed (e.g., number of *epidemic* months predicted relative to the total number of *epidemic* months observed). Specificity (true negative rate) measures how often a category is correctly discarded conditioned on the total number of times that category was not observed (e.g., number of non‐*epidemic* months predicted relative to the total number of non‐*epidemic* months). Each metric is measured from 0 to 1, with 1 representing a perfect score.

Random forest and negative binomial models vary in their categorical performances but tend to illustrate similar patterns in model skill across lead times. All categorical metrics tend to decline from short to long lead times. Accuracy for the random forest (negative binomial) models ranges from 0.71 to 0.85 (0.67–0.86), and specificity ranges from 0.71 to 0.89 (0.69–0.95) across all categories and lead times. Accuracy and specificity of the *epidemic* category experience the largest declines from 1‐ to 6‐month leads, compared to other categories. This suggests that the *epidemic* category is particularly susceptible to false positives at longer lead times, or overpredictions of DENV incidence. Sensitivity has the greatest variability among categorical metrics, ranging from 0.16 to 0.75 (0.19–0.68). On average, sensitivity is lowest in the *above safety* and *warning* categories. This implies that models are often predicting different categories when the observed category is between the two extremes (*below safety, epidemic*). Sensitivity is highest for the *epidemic* category, which is encouraging given that public health officials are likely most concerned with accurate predictions of high transmission scenarios.

Compared to predictions made by the reference models, the inclusion of climate information in the random forest model appears to make modest improvements. Across all cities, lead times, and categories, the random forest slightly improves in sensitivity (0.02), accuracy (0.04) and specificity (0.02) over the reference models, on average. These average improvements are dampened by the relatively poor performance of the random forest model compared to the reference model at the 1‐month lead. Nearly all increases in categorical skill come from the 3‐ and 6‐ month lead times. Average improvements by the negative binomial model are negligible (sensitivity: −0.01, specificity: 0.002, accuracy: −0.003). Given the modest performance of negative binomial models compared to random forest, the discussion below focuses largely on random forest results.

**Figure 6:**
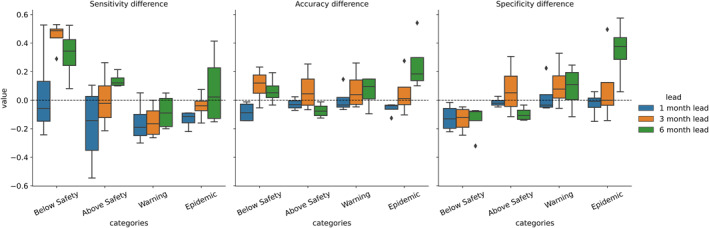
Difference between random forest and autoregressive model forecast sensitivity, accuracy, and specificity by epidemic channel category. Positive values indicate better performance of the random forest relative to the autoregressive model.

The scenarios in which the random forest model shows meaningful improvement over reference forecasts become more apparent when performance is broken down by category. The greatest improvements in accuracy and specificity are seen at 3‐ and 6‐ month leads for the *epidemic* category (Figure 6). Sensitivity improves most at 3‐ and 6‐ month leads for the *below safety* category. This may imply that the addition of climate information improves the detection of months in which transmission will be low, compared to reference models based solely on previous case data. The number of false predictions for epidemic months is also reduced, leading to improved specificity. This is largely driven by a decrease in the number of observed *below safety* events predicted to be *epidemic* events. It is also important to note that the inclusion of climate information sometimes results in a decrease in categorical skill compared to an autoregressive model. Declines are likely related to the choice of random forest for model construction, compared to linear regression for the autoregressive reference models.

**Figure 7:**
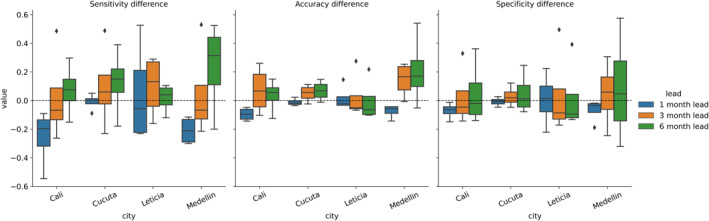
Difference between random forest and autoregressive model forecast sensitivity, accuracy, and specificity by city. Positive values indicate better performance of the random forest relative to the autoregressive model.

Model performance also varies by location. Categorical performance tends to decline slightly from the 1‐month lead to the 6‐month lead, however, accuracy and specificity remain high across lead times. Model accuracy ranges from 0.69 to 0.86 across all locations and lead times. Specificity falls between 0.77 and 0.9. While these scores are close, it is notable that Leticia models have the lowest accuracy and specificity across all lead times. Model sensitivities show a much larger range (0.29–0.67). The Cucuta models have the highest average sensitivity at each lead time. When comparing improvements in climate models over reference models, Cali, Cucuta, and Medellín show the greatest improvements at the 6‐month lead time (Figure 7). Leticia climate models generally show declining improvements over reference models from 1‐ to 6‐month lead times. Correlations between climate variables and DENV incidence in Leticia is generally lower than the other study locations, which may be reflected in the lack of climate model improvements at longer lead times.

### 3.3 Variable Importance

**Figure 8:**
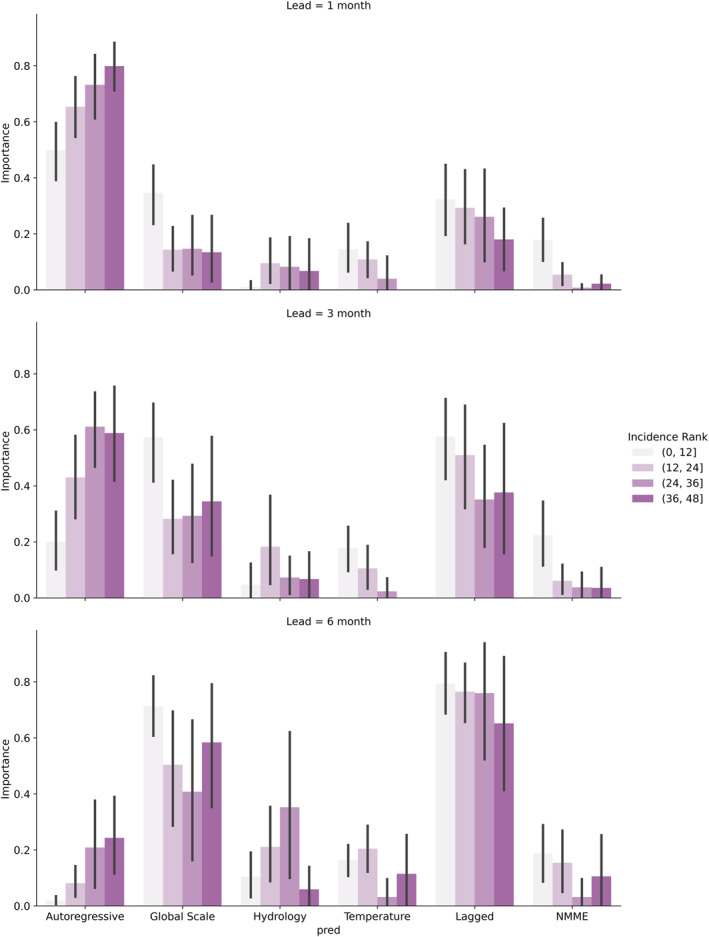
Average feature importance for each predictor category (see Table 2) at each lead time, binned by incidence rank. Low values correspond to low average dengue virus incidence and vice versa. Black bars represent a nonparametric 95% confidence interval calculated using the seaborn package.

Forecast performance may also vary depending on average DENV incidence. To evaluate the DENV conditions (high case load, low case load) under which climate information is most valuable for DENV prediction, variable importance of each predictor type is assessed for all random forest models at each lead time (all cities, months) (Figure 8). Variable importance is compared across a gradient of DENV conditions. To compare low case load conditions to high case load conditions, the boundary indicating the start of the *epidemic* category in each month and the city is ranked from lowest (1) to highest (48). This provides a proxy for average transmission conditions in each month and city. Average variable importance for each predictor category is compared across these ranks.

Autoregressive predictors are found to increase in importance from low transmission scenarios to high transmission scenarios. Conversely, nearly all categories of hydroclimate predictors decrease in importance from low to high transmission scenarios. This suggests that hydroclimate predictors are more effective in environments (cities and months) where DENV incidence is lower, based on long‐term averages, and autoregressive predictors perform better when DENV incidence is expected to be high. This pattern holds across lead times.

Random forest models that outperform reference models also tend to have lower DENV incidence on average. Skillful city and month‐specific random forest models (based on *R* ^2^) have a median incidence rank of 18. Models that were outperformed by reference models have a median incidence rank of 26. Categorical performance of random forest models also indicates that climate information improves skill for prediction of low DENV incidence, given the increases in sensitivity for the *below safety* category and increases in specificity for *epidemic* categories, particularly at longer leads.

## 4 Discussion

### 4.1 Lead Time Characteristics

Climate‐based forecasts for DENV incidence at multiple lead times provides insight into the potential for skillful prediction of DENV conditions and specific predictors contributing skill. Of the city and month‐specific random forest models that improve upon reference models, most are at 3‐ and 6‐ month lead times. This suggests the limited ability of climate variables to improve upon the predictive signal of autoregressive variables at the 1‐month lead. Random forest *R* ^2^ outperformed autoregressive models in six cases at the 1‐month lead time. In most of these cases, the autoregressive models had poor performance relative to average autoregressive skill at the 1‐month lead time (the average *R* ^2^ score for these autoregressive models is 0.45, compared to an average *R* ^2^ of 0.75 for autoregressive models at the 1‐month lead overall.) Autoregressive predictors still had the highest variable importance in all but one case.

Given the strong performance of autoregressive models at the 1‐month lead, climate‐based S2S DENV forecast efforts are likely best suited to longer lead times. At 3‐ and 6‐ month leads several autoregressive models perform worse than a naïve forecast using the long‐term average of DENV incidence. Autoregression works well at short lead times due to the typical structure of a dengue outbreak in the data. Cases typically rise, peak, and fall over several weeks. This pattern can be captured by using case counts at a 1‐month lead but tends to break down at longer lead times. In these scenarios, climate‐based models are particularly well positioned to make meaningful improvements in season‐ahead DENV prediction. At the 3‐ and 6‐month leads, 54% of autoregressive models perform equal to or worse than a naïve model, measured by *R* ^2^ (52 instances of 96 models). In these instances, random forest models conditioned on climate variables show notable skill in DENV prediction, with an average *R* ^2^ of 0.34 (0–0.61) and 0.19 (−0.75 – 0.71). This analysis shows that longer seasonal lead times, in locations with low autocorrelation, have the potential to benefit significantly from the development of climate‐based DENV forecasting models. When comparing by CRPSS, the random forest models show small but meaningful improvements over autoregressive models at longer lead times, with 64% of 6‐month lead predictions outperforming a naïve model, and 56% outperforming autoregressive models. This is a promising result for development of longer range DENV forecasts. While 3‐ and 6‐ month forecasts may not ultimately achieve the skill of the 1‐month forecasts, the predictive power from hydroclimatic conditions at seasonal timescales is meaningful. Seasonal forecasts of DENV provide information for a complimentary set of actions to the 1‐month lead forecasts (White et al., 2017). Timely large‐scale interventions are key to reducing the impacts of dengue outbreaks on human health and economies (Constenla et al., 2015). As discussed previously there are a variety of dengue prevention strategies, many of which revolve around protecting against mosquito bites or distributing vaccines (Wong et al., 2022). Whether the intervention involves habitat destruction, Wolbachia release, or vaccine distribution, large‐scale interventions are often time‐intensive and require planning and implementation over several months (Achee et al., 2015; Utarini et al., 2021; Wong et al., 2022). 3‐ and 6‐ month lead forecasts complement shorter lead forecasts by giving decision makers information with enough time to set large scale interventions in motion. While some of the improvements in 3‐ and 6‐ month forecast skill are modest, the predictive power of hydroclimate variables at these leads shows promise for development of operational season‐ahead DENV forecasts.

Analysis of multiple lead times also provides a unique opportunity to assess the performance of predictors at different time scales. As expected, the performance of autoregressive predictors tends to decline as lead time increases. Global climate predictors, including SSTs in ENSO and other regions and geopotential height, represent the largest importance and retention across lead times. This is driven largely by the importance of lagged regions of selected SST regions. ENSO also contributes to this increase, both through the inclusion of lagged ENSO indices and GFDL SPEAR forecast outputs. This is particularly pronounced at the 6‐month lead time, when the importance of autoregressive predictors is lowest. SST predictors are also likely to persist longer than many local variables (hydrology and temperature), which may contribute to their increased importance at longer lead times.

**Figure 9:**
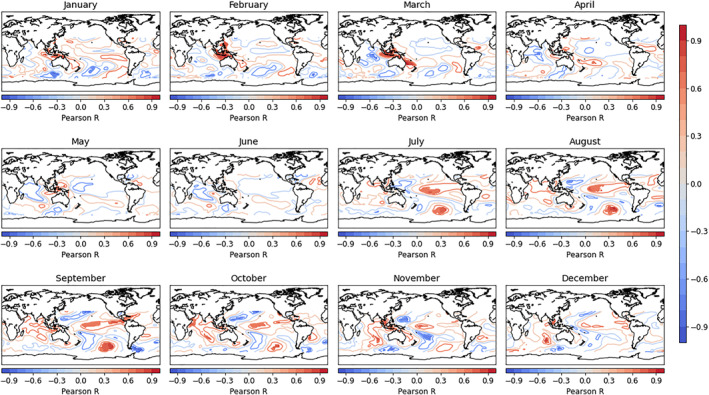
Correlations between sea surface temperatures (SSTs) (6‐month lag) and Medellín dengue virus (DENV) incidence. Months refer to SSTs (e.g., January SSTs correlated with July DENV incidence.) Correlations are indicated by contour lines. Regions selected as candidate predictors are filled.

Selected regions of SST tend to have higher average variable importance than ENSO indices. One likely reason is that these regions already capture much of the ENSO signal. For example, at the 6‐month lead time in Medellín, many of the selected SST regions fall within one of the defined ENSO regions (Figure 9). This is reflected in correlations with ENSO indices, where January–April DENV incidence is highly correlated with ENSO indices at a 6‐month lag (July‐October) (Figure 2). In addition to the ENSO signal, selected SST regions may capture additional teleconnections and increase the number of months in which SSTs have a measurable relationship with DENV incidence.

For example, regions associated with the Indian Ocean Dipole (IOD) show strong correlation from November – March with Medellín incidence. While ENSO is regarded as the dominant source of climatic variability in South America, some isolated impacts of the IOD on South American precipitation have been identified (Chan et al., 2008; Sena & Magnusdottir, 2021; Taschetto & Ambrizzi, 2012). Further, the IOD has been shown to be influenced by ENSO and alter its development in some cases (Annamalai et al., 2005; Zhang et al., 2015). Global analysis of SSTs captures these regions of influence that are otherwise missed by using ENSO indices alone.

While the utility of SSTs and geopotential height predictors are evident for all cities, the importance is consistently higher in Cali and Medellín. As discussed previously, these two cities have lower average annual temperatures that can act as a limiting factor for the spread of DENV. Given that temperature has strong concurrent correlations with DENV incidence in Cali and Medellín (Figure 1), it seems likely that the outsized influence of global climate predictors in these cities is related to the connection between ENSO and average temperature. For the study period, the average Pearson correlation between lagged monthly SSTs in the Nino 3.4 region and temperatures in Cali and Medellín are notable and persist as lead times increase (Table 4). Correlations are highest from November to March, when the ENSO phenomenon reaches its peak. Additionally, all cities show relatively strong autocorrelation at all lag times in average monthly temperature. This may partially explain the high retention rate of temperature predictors at even the 6‐month lead.

**Table 4:** Average (Min‐Max) Pearson Correlation by Month Between Nino 3.4 Sea Surface Temperatures and Mean Temperature

Lag	Cali	Medellín
1‐month	0.71 (0.43–0.96)	0.68 (0.40–0.91)
3‐month	0.67 (0.19–0.91)	0.62 (0.02–0.97)
6‐month	0.49 (−0.11 to 0.86)	0.42 (−0.35 to 0.83)

Hydrologic predictors appear to have a more complicated relationship with DENV incidence. Except for Leticia, most models include hydrologic predictors in less than half of the models at all lead times. The variables with the highest average importance across models are mean and max relative humidity. This is driven largely by the Leticia models, in which relative humidity represents most of the hydrologic variable importance. Cali and Medellín models include humidity as well, but only in three models across the two cities, with low (<15%) importance. Precipitation has some variable importance in eight of the 48 models; however, importance is inconsistent among locations, months, and lead times. As discussed previously precipitation can have interacting and contrasting effects on vector development and contact with humans. Therefore, the limited and inconsistent importance of precipitation in predicting DENV incidence is not necessarily surprising. Streamflow was also included as a predictor in Leticia but was not found to correlate significantly with DENV incidence at any month or lead time.

### 4.2 Dengue Conditions

In addition to an assessment of lead time performance, DENV forecasts may contribute a better understanding of the conditions under which climate‐based forecasts perform best. Categorical performance indicates that the addition of climate information tends to improve accurate forecasts of low DENV incidence categories. Comparing categorical results between random forest and autoregressive models reveals large increases in the number of *below safety* events that are correctly predicted at the 3‐ and 6‐ month leads, with an 84% and 100% (6‐month autoregressive has no accurate *below safety* predictions) increase at each lead, respectively. These gains appear to come from better discrimination of extremely high and low dengue conditions at the 3‐ and 6‐ month lead times (Figure 5). This is most apparent in the number of *below safety* months mis‐categorized as *epidemic* by the autoregressive models. At the 3‐ and 6‐month leads, using random forest models leads to these mis‐categorizations declining by 42% and 43%. This points to a weakness of autoregressive models in capturing low DENV incidence years.

An analysis of variable importance reinforces these results. When comparing average DENV conditions (as incidence rank) to variable importance, it becomes clear that climate variables are more important in the cities and months in which DENV incidence is typically low, and autoregressive predictors are more important when DENV incidence is high (Figure 6). This also suggests that climate‐based models perform best in scenarios where average DENV incidence is low. To verify this finding, incidence rank is compared to the percent of categorical predictions corrected by the random forest model over the reference model. Using the same groupings for incidence rank discussed above, at the 3‐ and 6‐ month lead, improvements in categorical forecast performance are concentrated in the cities and months with low average DENV caseload, largely driven by better performance in the *below safety* category. For both leads, some notable improvements are also seen in both the cities and months with the highest average DENV case load, driven by a reduction in the number of *below safety* events categorized as *epidemic* events.

**Figure 10:**
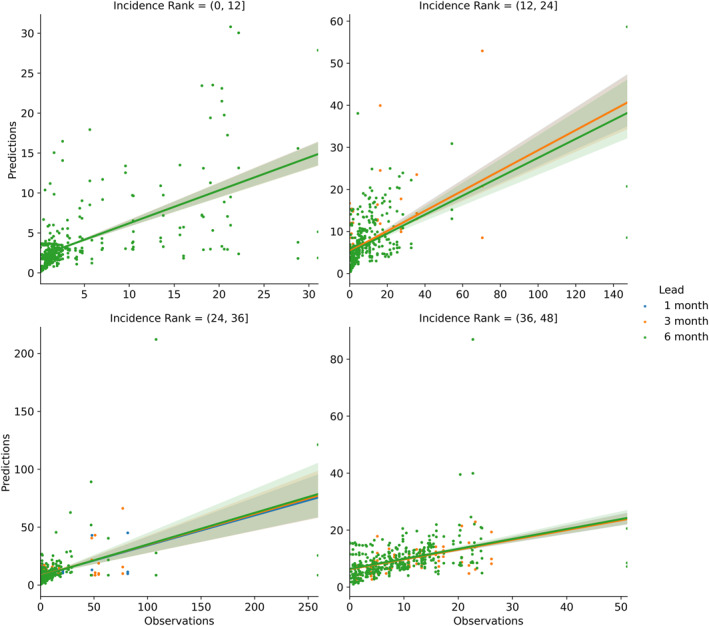
Autoregressive relationships for all cities, grouped by lead time and incidence rank. Linear models are fit to each lead in each incidence rank grouping.

One possible explanation for these shifts in variable importance is that autoregressive relationships in locations and months with lower average DENV incidence are poorly defined and have large uncertainties (Figure 10). Comparatively, the locations and months with high average incidence have far more well‐defined relationships. Locations and months in which autoregressive relationships perform poorly appear to be better positioned to benefit from the integration of climate information. In other words, advanced notice of environmental conditions that are favorable to DENV outbreaks is more useful when elevated DENV incidence is rare.

Together, the results of this modeling effort suggest that integration of climate information into DENV forecasts is particularly effective for accurate prediction of DENV incidence at 3‐ and 6‐ month lead times. This appears to be related to the declining performance of autoregressive models in DENV incidence at the 3‐ and 6‐ month lead times. The largest improvements are seen most frequently in the cities and months that experience low incidence on average, likely because climate information makes up for poor autoregressive predictions in these scenarios. In these places, seasonal advanced notice of a rise in dengue cases has strong potential to improve public health preparedness. As discussed previously, common prevention measures include distribution of vaccines, use of larvicide and insecticide, and promotion of personal protection from bites. With advanced notice of outbreak conditions public health officials can allocate funding, prepare supplies, and hire staff to implement vector‐control strategies. This information may be particularly useful in low‐incidence scenarios, where resources may be less readily available when an outbreak occurs. Future work is needed to investigate this result across a larger range of locations and average DENV case load conditions. Some benefit is also seen from climate‐based models in locations with high average DENV incidence, due to correction of extreme categorical misses. While some improvements are seen in the prediction of *epidemic* conditions, they are more modest.

### 4.3 Limitations

While this analysis provides some insights on the use of hydroclimate information in extended forecasts of DENV, it is important to note that this analysis takes place in only four cities. This analysis does not explicitly model the spatial relationships between environmental variables and DENV cases and results indicate that dengue case load and seasonality are variable among cities. Statistical relationships between DENV cases and hydroclimate predictors vary among cities as well. Future work is needed to investigate the transferability of this seasonal forecasting approach in a larger variety of locations.

## 5 Conclusions

In this paper, tailored statistical forecasting models for DENV incidence are developed at 1‐, 3‐, and 6‐ month lead times for each month in four cities across Colombia (Cali, Cúcuta, Medellín, and Leticia) to inform public health decision‐making at seasonal lead times. Forecast models are built using negative binomial and random forest structures and conditioned on autoregressive and hydroclimatic variables. A purely autoregressive model is compared as a null. Hydroclimate variables include global climate features (sea surface temperatures and geopotential height) as well as local temperature, precipitation, and relative humidity. Development of forecasts provides an opportunity to evaluate the relative contribution of hydroclimate variables to DENV predictions, lead times at which climate‐based forecasts are superior to autoregressive models, and the conditions (e.g., incidence severity) under which DENV forecasts perform best.

Random forest models outperform negative binomial models in most cases. Therefore, results from random forest models are primarily discussed. At the 1‐month lead, autoregressive predictors are the dominant source of predictive skill, included in 100% of models, and are rarely outperformed by climate variables. Autoregressive predictors are included in fewer models at the 3‐month and 6‐month leads, when global regions of SST and ENSO indices contribute most to DENV incidence prediction, followed by temperature. These predictors indicate the importance of temperature range in DENV case load.

Given the increased importance of hydroclimate predictors at 3‐ and 6‐month lead times, and decreasing autoregressive skill, forecasts are found to outperform autoregressive null models more frequently at these lead times. 25 (54%) models improve over null models at the 3‐month lead time, and 33 (70%) models improve over null models at the 6‐month lead. Unsurprisingly, the magnitude of improvement is greatest in city and month‐specific models where autoregressive variables have no predictive skill. Categorical accuracy ranges from 0.71 to 0.85, and specificity ranges from 0.71 to 0.89 across all categories and lead times. Sensitivity has the greatest variability among categorical metrics, ranging from 0.16 to 0.75. Improvements in categorical metrics over the null model are concentrated in the *below safety* and *epidemic* categories, driven by a reduction in mis‐categorizations of *below safety* as an *epidemic*.

An analysis of variable importance under different average DENV incidences reveals that autoregressive predictors are more important in cities and months with the highest average incidence. In the cities and months with low average DENV incidence, variable importance is highest among hydroclimate variables.

Given these results, three conclusions are drawn:

1. Climate‐based models are particularly skillful at 3‐ and 6‐ month lead times when autoregressive models often fail.
2. Regions of global SST, predominantly ENSO regions, provide the strongest source of predictive skill at 3‐ and 6‐ month leads.
3. Integrating climate information is most useful in scenarios (locations and months) in which average DENV incidence is low, autoregressive relationships are weak, but outbreaks may still occur.

Climate‐based DENV incidence forecasts have the potential to better inform public health interventions, particularly at seasonal lead times. Careful consideration of the strengths of such forecasts under different climatic and public health scenarios can help target dengue forecast development to improve public health preparedness and response.

## Global Research Collaboration Statement

We would like to acknowledge collaborative support and expertise provided by the Global Health Institute's One Health Center‐Colombia, in the conceptualization and execution of this work.

## Conflict of Interest

The authors declare no conflicts of interest relevant to this study.

## Data Availability

Climate and hydrology data used in this study is available publicly. DENV case data is accessed from SIVIGILA run by the National Institutes of Health of Colombia (https://portalsivigila.ins.gov.co). Codes for this work can be found on GitHub at https://github.com/mrwbeal/ColombiaS2SDengue. An archived version of this code is available at https://doi.org/10.5281/zenodo.14577189 (Beal, 2024).

## References

[gh270049-bib-0001] Abiodun, G. J. , Makinde, O. S. , Adeola, A. M. , Njabo, K. Y. , Witbooi, P. J. , Djidjou‐Demasse, R. , & Botai, J. O. (2019). A dynamical and zero‐inflated negative binomial regression modelling of malaria incidence in Limpopo Province, South Africa. International Journal of Environmental Research and Public Health, 16(11), 2000. 10.3390/ijerph16112000 31195637 PMC6603991

[gh270049-bib-0002] Achee, N. L. , Gould, F. , Perkins, T. A. , Reiner, R. C. , Morrison, A. C. , Ritchie, S. A. , et al. (2015). A critical assessment of vector control for dengue prevention. PLoS Neglected Tropical Diseases, 9(5), e0003655. 10.1371/journal.pntd.0003655 25951103 PMC4423954

[gh270049-bib-0003] Annamalai, H. , Xie, S. P. , McCreary, J. P. , & Murtugudde, R. (2005). Impact of Indian Ocean sea surface temperature on developing El Niño. Journal of Climate, 18(2), 302–319. 10.1175/jcli-3268.1

[gh270049-bib-0004] Baharom, M. , Ahmad, N. , Hod, R. , & Abdul Manaf, M. R. (2022). Dengue early warning system as outbreak prediction tool: A systematic review. Risk Management and Healthcare Policy, 15, 871–886. 10.2147/rmhp.s361106 35535237 PMC9078425

[gh270049-bib-0005] Barnston, A. G. (1994). Linear statistical short‐term climate predictive skill in the Northern Hemisphere. Journal of Climate, 7(10), 1513–1564. 10.1175/1520-0442(1994)007<1513:lsstcp>2.0.co;2

[gh270049-bib-0006] Beal, M. (2024). mrwbeal/ColombiaS2SDengue: Colombia S2S Dengue code archive. Zenodo. 10.5281/zenodo.14577190

[gh270049-bib-0007] Becker, E. , van den Dool, H. , & Zhang, Q. (2014). Predictability and forecast skill in NMME. Journal of Climate, 27(15), 5891–5906. 10.1175/jcli-d-13-00597.1

[gh270049-bib-0008] Bortman, M. (1999). Elaboración de corredores o canales endémicos mediante planillas de cálculo. Revista Panamericana de Salud Pública, 5, 1–8. 10.1590/s1020-49891999000100001 10050608

[gh270049-bib-0009] Breiman, L. (2001). Random forests. Machine Learning, 45(1), 5–32. 10.1023/a:1010933404324

[gh270049-bib-0010] Brunet, G. , Shapiro, M. , Hoskins, B. , Moncrieff, M. , Dole, R. , Kiladis, G. N. , et al. (2010). Collaboration of the weather and climate communities to advance subseasonal‐to‐seasonal prediction. Bulletin of the American Meteorological Society, 91(10), 1397–1406. 10.1175/2010bams3013.1

[gh270049-bib-0011] Cabrera, C. V. P. , & Selvaraj, J. J. (2020). Geographic shifts in the bioclimatic suitability for Aedes aegypti under climate change scenarios in Colombia. Heliyon, 6(1), e03101. 10.1016/j.heliyon.2019.e03101 31909268 PMC6940634

[gh270049-bib-0012] Cano‐Pérez, E. , Loyola, S. , Malambo‐García, D. , & Gómez‐Camargo, D. (2022). Climatic factors and the incidence of dengue in Cartagena, Colombian Caribbean Region. Revista da Sociedade Brasileira de Medicina Tropical, 55, e0072‐2022. 10.1590/0037-8682-0072-2022 36197377 PMC9536800

[gh270049-bib-0013] Chan, S. C. , Behera, S. K. , & Yamagata, T. (2008). Indian Ocean dipole influence on South American rainfall. Geophysical Research Letters, 35(14), L14S12. 10.1029/2008gl034204

[gh270049-bib-0014] Claypool, A. L. , Brandeau, M. L. , & Goldhaber‐Fiebert, J. D. (2021). Prevention and control of dengue and chikungunya in Colombia: A cost‐effectiveness analysis. PLoS Neglected Tropical Diseases, 15(12), e0010086. 10.1371/journal.pntd.0010086 34965277 PMC8752007

[gh270049-bib-0015] Constenla, D. , Garcia, C. , & Lefcourt, N. (2015). Assessing the economics of dengue: Results from a systematic review of the Literature and expert survey. PharmacoEconomics, 33(11), 1107–1135. 10.1007/s40273-015-0294-7 26048354

[gh270049-bib-0016] DREF . (2013). 2013 DREF final report Colombia: Dengue outbreak. Retrieved from https://reliefweb.int/report/colombia/colombia‐dengue‐dref‐final‐report‐n‐mdrco010

[gh270049-bib-0017] DREF . (2019). 2019 DREF final report Colombia: Dengue outbreak. Retrieved from https://reliefweb.int/report/colombia/colombia‐dengue‐outbreak‐dref‐operation‐no‐mdrco016‐final‐report

[gh270049-bib-0018] Duarte, J. L. , Diaz‐Quijano, F. A. , Batista, A. C. , & Giatti, L. L. (2019). Climatic variables associated with dengue incidence in a city of the Western Brazilian Amazon region. Revista da Sociedade Brasileira de Medicina Tropical, 52, e20180429. 10.1590/0037-8682-0429-2018 30810657

[gh270049-bib-0019] Eastin, M. D. , Delmelle, E. , Casas, I. , Wexler, J. , & Self, C. (2014). Intra‐and interseasonal autoregressive prediction of dengue outbreaks using local weather and regional climate for a tropical environment in Colombia. The American Journal of Tropical Medicine and Hygiene, 91(3), 598–610. 10.4269/ajtmh.13-0303 24957546 PMC4155567

[gh270049-bib-0020] Epstein, E. S. (1969). A scoring System for probability forecasts of ranked categories. Journal of Applied Meteorology and Climatology, 8(6), 985–987. 10.1175/1520-0450(1969)008<0985:ASSFPF>2.0.CO;2

[gh270049-bib-0021] Evans, O. P. , & Adenomon, M. O. (2014). Modeling the prevalence of malaria in Niger State: An application of Poisson regression and negative binomial regression models. International Journal of the Physical Sciences, 2, 61–68.

[gh270049-bib-0022] Giannini, A. , Kushnir, Y. , & Cane, M. A. (2000). Interannual variability of Caribbean rainfall, ENSO, and the Atlantic Ocean. Journal of Climate, 13(2), 297–311. 10.1175/1520-0442(2000)013<0297:ivocre>2.0.co;2

[gh270049-bib-0023] Gubler, D. J. (2011). Dengue, urbanization and globalization: The unholy trinity of the 21st century. Tropical Medicine and Health, 39(4SUPPLEMENT), S3–S11. 10.2149/tmh.2011-s05 PMC3317603 22500131

[gh270049-bib-0024] Gutierrez‐Barbosa, H. , Medina‐Moreno, S. , Zapata, J. C. , & Chua, J. V. (2020). Dengue infections in Colombia: Epidemiological trends of a hyperendemic country. Tropical Medicine and Infectious Disease, 5(4), 156. 10.3390/tropicalmed5040156 33022908 PMC7709707

[gh270049-bib-0025] Guzman, M. G. , Halstead, S. B. , Artsob, H. , Buchy, P. , Farrar, J. , Gubler, D. J. , et al. (2010). Dengue: A continuing global threat. Nature Reviews Microbiology, 8(Suppl 12), S7–S16. 10.1038/nrmicro2460 21079655 PMC4333201

[gh270049-bib-0026] Helsel, D. R. , & Hirsch, R. M. (1992). Statistical methods in water resources (Vol. 49). Elsevier.

[gh270049-bib-0027] Hersbach, H. (2000). Decomposition of the continuous ranked probability score for ensemble prediction systems. Weather and Forecasting, 15(5), 559–570. 10.1175/1520-0434(2000)015<0559:dotcrp>2.0.co;2

[gh270049-bib-0028] Hilbe, J. M. (2011). Negative binomial regression. Cambridge University Press.

[gh270049-bib-0029] Hunter, P. R. (2003). Climate change and waterborne and vector‐borne disease. Journal of Applied Microbiology, 94(s1), 37–46. 10.1046/j.1365-2672.94.s1.5.x 12675935

[gh270049-bib-0030] Johansson, M. A. , Dominici, F. , & Glass, G. E. (2009). Local and global effects of climate on dengue transmission in Puerto Rico. PLoS Neglected Tropical Diseases, 3(2), e382. 10.1371/journal.pntd.0000382 19221592 PMC2637540

[gh270049-bib-0031] Jones, K. E. , Patel, N. G. , Levy, M. A. , Storeygard, A. , Balk, D. , Gittleman, J. L. , & Daszak, P. (2008). Global trends in emerging infectious diseases. Nature, 451(7181), 990–993. 10.1038/nature06536 18288193 PMC5960580

[gh270049-bib-0032] Kirtman, B. P. , Min, D. , Infanti, J. M. , Kinter, J. L. , Paolino, D. A. , Zhang, Q. , et al. (2014). The North American multimodel ensemble: Phase‐1 seasonal‐to‐interannual prediction; phase‐2 toward developing intraseasonal prediction. Bulletin of the American Meteorological Society, 95(4), 585–601. 10.1175/bams-d-12-00050.1

[gh270049-bib-0033] Lancet, T. (2024). Dengue: The threat to health now and in the future. The Lancet, 404(10450), 311. 10.1016/S0140-6736(24)01542-3 39067890

[gh270049-bib-0034] Lenharo, M. (2023). Dengue is breaking records in the Americas‐what’s behind the surge? Nature. 10.1038/d41586-023-02423-w 37500998

[gh270049-bib-0035] Lenters, J. D. , & Cook, K. H. (1997). On the origin of the Bolivian high and related circulation features of the South American climate. Journal of the Atmospheric Sciences, 54(5), 656–678. 10.1175/1520-0469(1997)054<0656:otootb>2.0.co;2

[gh270049-bib-0036] Markowski, G. R. , & North, G. R. (2003). Climatic influence of sea surface temperature: Evidence of substantial precipitation correlation and predictability. Journal of Hydrometeorology, 4(5), 856–877. 10.1175/1525-7541(2003)004<0856:CIOSST>2.0.CO;2

[gh270049-bib-0037] Me, W. , Hamilton, D. P. , McBride, C. G. , Abell, J. M. , & Hicks, B. J. (2018). Modelling hydrology and water quality in a mixed land use catchment and eutrophic lake: Effects of nutrient load reductions and climate change. Environmental Modelling & Software, 109, 114–133. 10.1016/j.envsoft.2018.08.001

[gh270049-bib-0038] Muñoz, Á. G. , Chourio, X. , Rivière‐Cinnamond, A. , Diuk‐Wasser, M. A. , Kache, P. A. , Mordecai, E. A. , et al. (2020). Ae DES: A next‐generation monitoring and forecasting system for environmental suitability of Aedes‐borne disease transmission. Scientific Reports, 10(1), 12640. 10.1038/s41598-020-69625-4 32724218 PMC7387552

[gh270049-bib-0039] Muñoz, E. , Poveda, G. , Arbeláez, M. P. , & Vélez, I. D. (2021). Spatiotemporal dynamics of dengue in Colombia in relation to the combined effects of local climate and ENSO. Acta Tropica, 224, 106136. 10.1016/j.actatropica.2021.106136 34555353

[gh270049-bib-0040] Ocampo, C. B. , Mina, N. J. , Carabalí, M. , Alexander, N. , & Osorio, L. (2014). Reduction in dengue cases observed during mass control of Aedes (Stegomyia) in street catch basins in an endemic urban area in Colombia. Acta Tropica, 132, 15–22. 10.1016/j.actatropica.2013.12.019 24388794 PMC4654410

[gh270049-bib-0041] Ordonez‐Sierra, G. , Sarmiento‐Senior, D. , Gomez, J. F. J. , Giraldo, P. , Ramírez, A. P. , & Olano, V. A. (2021). Multilevel analysis of social, climatic and entomological factors that influenced dengue occurrence in three municipalities in Colombia. One Health, 12, 100234. 10.1016/j.onehlt.2021.100234 33855157 PMC8025047

[gh270049-bib-0042] Pan American Health Organization . (2024). Summary of the situation by subregion 1 subregion of the central American Isthmus and Mexico. Retrieved from https://www3.paho.org/data/index.php/en/mnu‐topics/indicadores‐dengue‐en.html

[gh270049-bib-0043] Pedregosa, F. , Varoquaux, G. , Gramfort, A. , Michel, V. , Thirion, B. , Grisel, O. , et al. (2011). Scikit‐learn: Machine learning in Python. Journal of Machine Learning Research, 12, 2825–2830.

[gh270049-bib-0044] Pegion, K. , Kirtman, B. P. , Becker, E. , Collins, D. C. , LaJoie, E. , Burgman, R. , et al. (2019). The Subseasonal Experiment (SubX): A multimodel subseasonal prediction experiment. Bulletin of the American Meteorological Society, 100(10), 2043–2060. 10.1175/bams-d-18-0270.1

[gh270049-bib-0045] Poveda, G. , Alvarez, D. M. , & Rueda, O. A. (2011). Hydro‐climatic variability over the Andes of Colombia associated with ENSO: A review of climatic processes and their impact on one of the Earth’s most important biodiversity hotspots. Climate Dynamics, 36(11–12), 2233–2249. 10.1007/s00382-010-0931-y

[gh270049-bib-0046] Poveda, G. , Graham, N. E. , Epstein, P. R. , Rojas, W. , Quiñones, M. L. , Velez, I. D. , & Martens, W. J. M. (2000). Climate and ENSO variability associated with vector‐borne diseases in Colombia. El Niño and the Southern Oscillation, Multiscale Variability and Global and Regional Impacts, 1, 183–204.

[gh270049-bib-0047] Rico‐Mendoza, A. , Alexandra, P.‐R. , Chang, A. , Encinales, L. , & Lynch, R. (2019). Co‐circulation of dengue, chikungunya, and Zika viruses in Colombia from 2008 to 2018. Revista Panamericana de Salud Pública, 43, 1. 10.26633/rpsp.2019.49 PMC6548069 31171921

[gh270049-bib-0048] Salas, H. D. , Poveda, G. , Mesa, Ó. J. , & Marwan, N. (2020). Generalized synchronization between ENSO and hydrological variables in Colombia: A recurrence quantification approach. Frontiers in Applied Mathematics and Statistics, 6, 3. 10.3389/fams.2020.00003

[gh270049-bib-0049] Sena, A. C. T. , & Magnusdottir, G. (2021). Influence of the Indian Ocean dipole on the large‐scale circulation in South America. Journal of Climate, 34(15), 6057–6068. 10.1175/jcli-d-20-0669.1

[gh270049-bib-0050] Sepulveda, L. S. , & Vasilieva, O. (2016). Optimal control approach to dengue reduction and prevention in Cali, Colombia. Mathematical Methods in the Applied Sciences, 39(18), 5475–5496. 10.1002/mma.3932

[gh270049-bib-0051] Taschetto, A. S. , & Ambrizzi, T. (2012). Can Indian Ocean SST anomalies influence South American rainfall? Climate Dynamics, 38(7–8), 1615–1628. 10.1007/s00382-011-1165-3

[gh270049-bib-0052] Tompkins, A. M. , Lowe, R. , Nissan, H. , Martiny, N. , Roucou, P. , Thomson, M. C. , & Nakazawa, T. (2019). Predicting climate impacts on health at sub‐seasonal to seasonal timescales. In Sub‐seasonal to seasonal prediction (pp. 455–477). Elsevier.

[gh270049-bib-0053] Umaña, J. D. , Montenegro‐Torres, J. , & Otero, J. (2024). epiCo: Provides statistical and visualization tools for the analysis of outbreaks of vector‐borne diseases (VBDs) in Colombia.

[gh270049-bib-0054] Utarini, A. , Indriani, C. , Ahmad, R. A. , Tantowijoyo, W. , Arguni, E. , Ansari, M. R. , et al. (2021). Efficacy of Wolbachia‐infected mosquito deployments for the control of dengue. New England Journal of Medicine, 384(23), 2177–2186. 10.1056/NEJMoa2030243 34107180 PMC8103655

[gh270049-bib-0055] Villar, L. A. , Rojas, D. P. , Besada‐Lombana, S. , & Sarti, E. (2015). Epidemiological trends of dengue disease in Colombia (2000–2011): A systematic review. PLoS Neglected Tropical Diseases, 9(3), e0003499. 10.1371/journal.pntd.0003499 25790245 PMC4366106

[gh270049-bib-0056] Villegas, J. G. , Gutiérrez, E. V. , Barrera Ferro, D. , Muriel, O. , Felipe, A. , Paredes Bayona, J. E. , et al. (2020). Aplicaciones de investigación de operaciones en sistemas de salud en Colombia. Editorial Pontificia Universidad Javeriana.

[gh270049-bib-0057] Wager, S. , Hastie, T. , & Efron, B. (2014). Confidence intervals for random forests: The jackknife and the infinitesimal jackknife. Journal of Machine Learning Research, 15(1), 1625–1651. 25580094 PMC4286302

[gh270049-bib-0058] Waylen, P. , & Poveda, G. (2002). El Niño‐Southern oscillation and aspects of western South American hydro‐climatology. Hydrological Processes, 16(6), 1247–1260. 10.1002/hyp.1060

[gh270049-bib-0059] White, C. J. , Carlsen, H. , Robertson, A. W. , Klein, R. J. T. , Lazo, J. K. , Kumar, A. , et al. (2017). Potential applications of subseasonal‐to‐seasonal (S2S) predictions. Meteorological Applications, 24(3), 315–325. 10.1002/met.1654

[gh270049-bib-0060] Whiting, E. C. , Khan, A. , & Gubler, W. D. (2001). Effect of temperature and water potential on survival and mycelial growth of *Phaeomoniella chlamydospora* and *Phaeoacremonium* spp. Plant Disease, 85(2), 195–201. 10.1094/pdis.2001.85.2.195 30831942

[gh270049-bib-0061] WHO . (2019). Dengue vaccine: WHO position paper, September 2018—Recommendations. Vaccine, 37(35), 4848–4849. 10.1016/j.vaccine.2018.09.063 30424888

[gh270049-bib-0062] Wong, J. M. , Adams, L. E. , Durbin, A. P. , Muñoz‐Jordán, J. L. , Poehling, K. A. , Sánchez‐González, L. M. , et al. (2022). Dengue: A growing problem with new interventions. Pediatrics, 149(6), e2021055522. 10.1542/peds.2021-055522 35543085

[gh270049-bib-0063] Yuan, H.‐Y. , Liang, J. , Lin, P.‐S. , Sucipto, K. , Tsegaye, M. M. , Wen, T.‐H. , et al. (2020). The effects of seasonal climate variability on dengue annual incidence in Hong Kong: A modelling study. Scientific Reports, 10(1), 4297. 10.1038/s41598-020-60309-7 32152334 PMC7062697

[gh270049-bib-0064] Zhang, W. , Wang, Y. , Jin, F. , Stuecker, M. F. , & Turner, A. G. (2015). Impact of different El Niño types on the El Niño/IOD relationship. Geophysical Research Letters, 42(20), 8570–8576. 10.1002/2015gl065703

[gh270049-bib-0065] Zhao, N. , Charland, K. , Carabali, M. , Nsoesie, E. O. , Maheu‐Giroux, M. , Rees, E. , et al. (2020). Machine learning and dengue forecasting: Comparing random forests and artificial neural networks for predicting dengue burden at national and sub‐national scales in Colombia. PLoS Neglected Tropical Diseases, 14(9), e0008056. 10.1371/journal.pntd.0008056 32970674 PMC7537891

[gh270049-bib-0066] Zimmerman, B. G. , Vimont, D. J. , & Block, P. J. (2016). Utilizing the state of ENSO as a means for season‐ahead predictor selection. Water Resources Research, 52(5), 3761–3774. 10.1002/2015wr017644

